# Inhibitors of NLRP3 Inflammasome Formation: A Cardioprotective Role for the Gasotransmitters Carbon Monoxide, Nitric Oxide, and Hydrogen Sulphide in Acute Myocardial Infarction

**DOI:** 10.3390/ijms25179247

**Published:** 2024-08-26

**Authors:** Fergus M. Payne, Alisha R. Dabb, Joanne C. Harrison, Ivan A. Sammut

**Affiliations:** Department of Pharmacology and Toxicology and HeartOtago, School of Biomedical Sciences, University of Otago, Dunedin 9054, New Zealand; fergus.payne@otago.ac.nz (F.M.P.); adabb@malaghan.org.nz (A.R.D.); joanne.harrison@otago.ac.nz (J.C.H.)

**Keywords:** NLRP3, carbon monoxide, nitric oxide, hydrogen sulphide

## Abstract

Myocardial ischaemia reperfusion injury (IRI) occurring from acute coronary artery disease or cardiac surgical interventions such as bypass surgery can result in myocardial dysfunction, presenting as, myocardial “stunning”, arrhythmias, infarction, and adverse cardiac remodelling, and may lead to both a systemic and a localised inflammatory response. This localised cardiac inflammatory response is regulated through the nucleotide-binding oligomerisation domain (NACHT), leucine-rich repeat (LRR)-containing protein family pyrin domain (PYD)-3 (NLRP3) inflammasome, a multimeric structure whose components are present within both cardiomyocytes and in cardiac fibroblasts. The NLRP3 inflammasome is activated via numerous danger signals produced by IRI and is central to the resultant innate immune response. Inhibition of this inherent inflammatory response has been shown to protect the myocardium and stop the occurrence of the systemic inflammatory response syndrome following the re-establishment of cardiac circulation. Therapies to prevent NLRP3 inflammasome formation in the clinic are currently lacking, and therefore, new pharmacotherapies are required. This review will highlight the role of the NLRP3 inflammasome within the myocardium during IRI and will examine the therapeutic value of inflammasome inhibition with particular attention to carbon monoxide, nitric oxide, and hydrogen sulphide as potential pharmacological inhibitors of NLRP3 inflammasome activation.

## 1. Introduction

Pharmacological or percutaneous revascularisation procedures are commonly applied clinical interventions used to rapidly restore coronary perfusion and salvage as much of the jeopardised myocardium as possible in acutely ischaemic hearts. In selected patients, coronary artery bypass graft (CABG) procedures can be advocated to improve coronary flow [1] to the damaged myocardium. Paradoxically, however, the reintroduction of oxygenated blood to the damaged myocardium in all these revascularisation procedures can result in ischaemia reperfusion injury (IRI) and provoke further risk of infarct development in the postoperative period. The incidence of adverse outcomes in CABG patients varies with general factors such as the duration of aortic cross-clamp time [2], patient age [3], gender, morbidity, and body weight [4]. Additionally, the increasingly common presence of underlying cardiac pathology, such as hypertrophy [5] resulting from hypertensive, diabetic or rheumatic heart diseases, or genetic aetiologies, remains a major contributing factor to poor outcomes [6]. Hence, increasing effort has been directed to the development of effective cardioprotective conditioning interventions to protect the myocardium against reperfusion injury and to improve revascularisation outcomes for high-risk patients with vulnerable hearts [7,8]. If unchecked, IRI can result in both systemic and innate localised inflammatory responses. This review will examine the current understanding of the role of the inherent localised NLRP3-regulated inflammatory response in myocardial injury and will assess the potential of the novel gaseous conditioning agents carbon monoxide (CO), nitric oxide (NO), and hydrogen sulphide (H_2_S) as effective inflammasome inhibitors.

## 2. Myocardial Ischaemia Reperfusion Injury (IRI)

The imposition of an ischaemic insult to the myocardium rapidly depletes oxygen availability to cardiomyocytes for mitochondrial β-oxidation so that cellular metabolism becomes dependent on anaerobic glycolysis [9,10]. Prolongation of the ischaemic episode, however, can lead to the myocardium becoming progressively acidotic (<pH 7) through the accumulation of lactate and H^+^, thereby exacerbating myocardial injury [11,12,13]. The resultant intracellular accumulation of protons in this acidotic environment activates the Na^+^/H^+^ antiporter, serving to elevate cytosolic Na^+^ and drive the Na^+^/Ca^2+^ exchanger to import more Ca^2+^ into the myocardial cells [13,14,15,16]. High cytosolic Ca^2+^ levels have been firmly associated with arrhythmia formation, contributing to myocardial dysfunction, morbidity and mortality after IRI [17,18]. Raised cytosolic Ca^2+^ levels are also sequestered by mitochondria. The resultant mitochondrial Ca^2+^ overload facilitates ischaemic injury via mitochondrial swelling and the activation of proteases such as calpains, which lead to cell death via activation of pro-apoptotic proteins [19,20,21,22]. Within minutes of reperfusion onset, the reactive oxygen species (ROS) formed through mitochondrial electron leak, in conjunction with elevated mitochondrial Ca^2+^ and intracellular pH normalisation towards physiological levels (pH 7.2), permit the opening of the non-selective mitochondrial permeable transition pore (mPTP) [23,24]. Prolonged opening of the mPTP catastrophically disrupts the mitochondrial electrochemical gradient, impairing adenosine triphosphate (ATP) production and releasing a burst of ROS and cytochrome c to initiate apoptotic and necrotic cell death processes [25,26,27,28]. These various cell death processes defined by distinct modes of pathway activation subsequentially permeate throughout the infarcted region of the myocardium, resulting in irreversible cardiac injury.

### Inflammatory Responses Following Myocardial IRI

Concurrent to the cardiomyocyte apoptosis, necrosis and pyroptosis events occurring in cardiac IRI, pro-inflammatory signalling cascades are simultaneously upregulated, which leads to long-term cardiac fibrosis, ventricular hypertrophy, and stenosis, rendering the heart susceptible to failure [29,30]. Previous research has mainly explored the impact of a systemic inflammatory response, occurring on reperfusion in cardiac surgery [31]. While this complement cascade-mediated injury is significant, there have been few investigations into the inherent inflammatory response that occurs within the myocardium during ischaemic clamping and reperfusion procedures [32]. By removing the cellular debris and dead cells and initiating tissue regeneration, this sterile inflammatory response forms an essential initial recovery process that has been suggested to salvage the infarcted myocardium allowing scar tissue to form [33]. These beneficial effects are considered to outweigh the associated adverse long-term inflammatory pathology, which if unattenuated or excessive, can cause extensive cell death leading to increased fibroblast activation [34,35]. The resulting infiltrative and reparative fibrotic scaring can produce a stiffened ventricular wall adversely impacting myocardial function and viability and ultimately reducing end-organ perfusion, with significant adverse clinical outcomes [35].

This innate ischaemic myocardial inflammatory response is initiated through the formation of multimeric inflammasome structures. A number of distinct inflammasome types have been identified, although only NLRP1 and NLRP3 have been indicated to be upregulated during cardiac ischaemia and reperfusion, acting as key contributors to myocardial IRI, with NLRP3 being the most extensively studied and recognised [34,36,37,38]. Activation of NLRP3 causes necrotic cell death and releases active pro-inflammatory cytokines that can recruit innate immune cells to the injury site [39]. The subsequent systemic inflammatory response syndrome is implicated in myocardial dysfunction and end-organ injury. Additionally, caspase-1 activation via NLRP3 can lead to the release of the pro-inflammatory protein, high mobility group box protein 1 (HMGB1), into the microenvironment, which can further exacerbate the injury within the myocardium and throughout the body [40]. Inhibition of NLRP3 activation within the isolated heart during bypass surgery may therefore serve to simultaneously prevent the formation of an intrinsic myocardial inflammatory cascade and the consequent development of a systemic inflammatory response syndrome.

## 3. The NLRP3 Inflammasome

**Figure 1:**
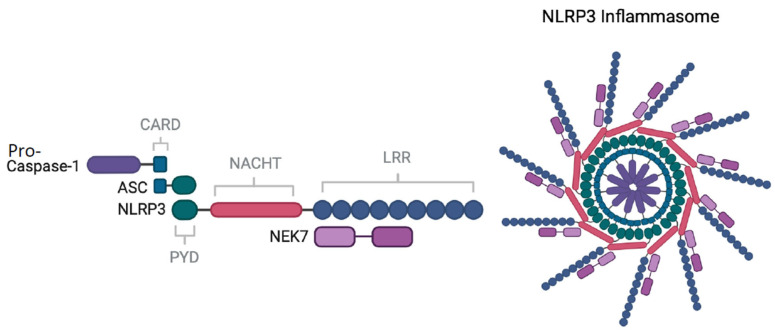
**Structure of the NLRP3 inflammasome.** Associations between NLRP3 and ASC occur via PYD-PYD interactions. NLRP3 does not have a CARD domain and requires ASC to interface with the CARD domain present on pro-caspase-1 using a CARD-CARD interaction. NEK7 interacts with the LRR domain on NLRP3. Multiple replicates of these interactions come together and assemble the NLRP3 inflammasome with pro-caspase 1 filaments branching out from the central core of polymerised ASC to cleave proteins.

The NLRP3 inflammasome (also referred to as NALP3 or cryopyrin) is a large multimeric protein complex (Figure 1) containing a central nucleotide-binding oligomerisation domain (NACHT), a C-terminus leucine-rich repeat (LRR) and an N-terminal pyrin domain (PYD) [41]. The PYD domain located at the N-terminal of NLRP3 facilitates the recruitment of the apoptosis-associated speck-like protein (ASC) through PYD-PYD interactions [42]. ASC also contains a caspase activation and recruitment domain (CARD) allowing the enlistment of pro-caspase-1 and hence activation into caspase-1 [43]. The NIMA (“never in mitosis gene a”)-related serine/threonine kinase 7 (NEK7) is a scaffolding protein consisting of a catalytic domain that interacts with the LRR domain of NLRP3 [44]. This interaction is proposed to break the inactive NLRP3 cage, transforming it into an active NLRP3 inflammasome disk [45].

### 3.1. NLRP3 Priming

**Figure 2:**
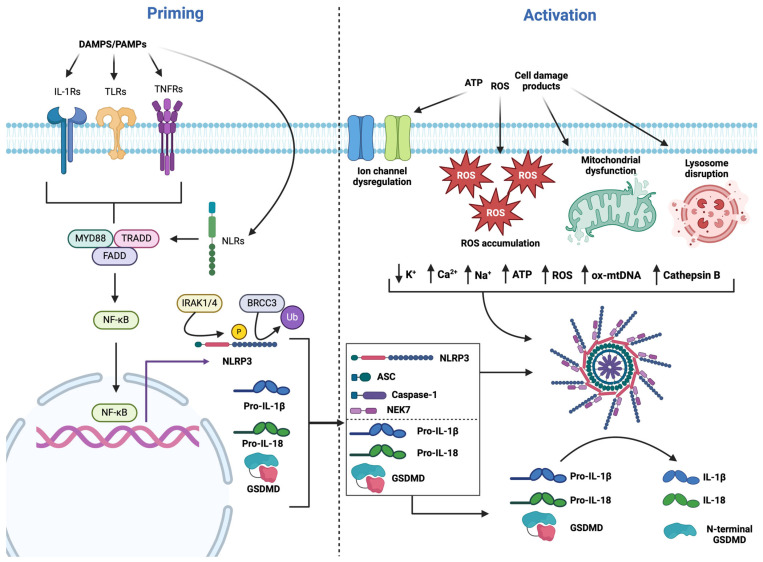
**Simplified illustration of the signalling pathway depicting priming and activation of the NLRP3 inflammasome.** PAMPs and DAMPs bind to their respective PRRs (IL-1Rs, TLRs, TNFRs, NLRs) to activate NF-κB, which upregulates the genes NLRP3, GSDMD, pro-IL-1β, and pro-IL18 within the cytosol. Concurrently, BRCC3 deubiquitinates NLRP3 and IRAK1/4 phosphorylates NLRP3 to also upregulate the priming of NLRP3 to aid inflammasome formation. Activation of NLRP3 is initiated by ion channel dysregulation, ROS accumulation, mitochondrial dysfunction, and lysosome disruption. Together, these generate key signals, including reduced K^+^ and increased Ca^2+^, Na^+^, ATP, ox-mtDNA, and cathepsin B to activate NLRP3 inflammasome formation. Activated inflammasomes will cleave pro-IL-1β, pro-IL18, and GSDMD to active IL-1β, IL-18, and the N-terminal of GSDMD to initiate pyroptosis.

Formation and activation of the NLRP3 inflammasome requires both priming and activation steps [46,47]. While cytosolic NLRP3 is normally expressed at low levels, priming of NLRP3 is needed to increase messenger ribonucleic acid levels within the cytosol to allow for inflammasome formation [48]. Additionally, baseline levels of cytosolic NLRP3 are rendered inactive via ubiquitination at the LRR domain [49,50]. In order for this priming event to be activated, pattern recognition receptors (PRRs) must first bind pathogen- and damage-associated molecular patterns (PAMPs and DAMPs) generated during tissue damage such as cellular debris, mitochondrial ROS, and ATP released into the cytosol [51,52]. DAMPs arising during IRI have been significantly linked to the development of irreversible changes within the myocardium, including apoptosis, fibrosis, and hypertrophy [32,37,39]. PRRs involved in this process include toll-like receptors (TLRs), nucleotide-binding oligomerisation domain-like receptors (NLRs), tumour necrosis factor receptors (TNFRs), and interleukin-1 receptors (IL-1Rs) (Figure 2) [49,53,54]. Activation of PRRs will in turn activate multiple adaptor proteins downstream of each receptor subtype, which can include either myeloid differentiation factor 88 (MyD88) or tumour necrosis factor 1 death domain protein (TRADD) dependent signalling [55,56] and subsequently lead to nuclear factor (NF)-κB activation [57,58,59]. In vitro and in vivo knockout (KO) studies have suggested that the adaptor FS-7-associated surface antigen-associated death domain protein (FADD) also facilitates NF-κB activation and is indispensable for NLRP3 priming [58,60].

NLRP3 inflammasome components have been identified in both cardiomyocyte and leukocytes as well as non-cardiomyocyte populations such as fibroblasts and endothelial cells within the mammalian heart [61]. However, as none of the components of the inflammasome are constitutively expressed within healthy cardiomyocytes, translocation of NF-κB to the nucleus is responsible for inducing the expression of genes for NLRP3, ASC, and pro-caspase 1, as well as for the substrates of activated caspase-1, pro-IL-1β, pro-IL-18, and gasdermin D (GSDMD) [49,62]. The increased cytosolic levels of these proteins allow NLRP3 inflammasome formation to occur (Figure 2) and subsequently, trigger an inflammatory response. NLRP3 inflammasome activation can also be primed via post-translational modifications; activation of the interleukin receptor-associated kinases 1/4 (IRAK1/4) phosphorylates the LRR domain of NLRP3 and promotes NLRP3-ASC interactions [63]. Non-transcriptional priming involving the lysine-63 deubiquitinate (BRCC3) mediates NLRP3 deubiquitylation of free NLRP3 at the LRR domain to also promote NLRP3-ASC interactions [48,50].

### 3.2. NLRP3 Activation

Prior to complete NLRP3 activation, a complex combination of cellular signalling events is required to allow NLRP3 inflammasome assembly and activation. The four most important activating signals in the context of IRI are generated through ion channel dysregulation [64], ROS accumulation [65], mitochondrial rupture [52], and lysosome disruption [66].

Ischaemia depletes intracellular ATP levels, resulting in ATP-gated ion channel dysregulation, facilitating cytosolic Na^+^ accumulation. Consequently, Na^+^ accumulation results in significant K^+^ efflux upon reperfusion [67]. He, et al. [68] reported that upon K^+^ efflux, NEK7 interacts with the LRR domain of NLRP3 to facilitate inflammasome assembly, which was abolished in NEK7^−/−^ KO cells. In addition, K^+^ efflux increases Ca^2+^-independent phospholipase A_2_ activation by approximately 75% in human monocytes, aiding in the cleavage of pro-IL-1β into active IL-1β [69]. While intracellular Ca^2+^ accumulation is considered insufficient to induce NLRP3 inflammasome activation [70], extracellular Ca^2+^ accumulation was established to activate the G-protein-coupled receptor class C group 6-member A to facilitate NLRP3 inflammasome activation [71].

Concurrent with ion channel dysregulation, ROS accumulation and release is a strong signal for NLRP3 activation as identified in several in vivo models of IRI [32,72,73]. ROS species can provoke mitochondrial dysfunction by stimulating the mPTP opening, resulting in mitochondrial swelling, and the release of mitochondrial ROS and oxidised mitochondrial DNA (ox-mtDNA) (Figure 2) [74]. While mitochondrial ROS can induce NLRP3 inflammasome activation, it is not the sole driver [75,76]. Instead, ox-mtDNA appears to be the most significant stimulus as it directly promotes NLRP3-ASC interactions through protein modifications on ASC, increasing the binding affinity for NLRP3 [74]. Furthermore, ox-mtDNA can also directly bind to the PYD of NLRP3 to facilitate inflammasome assembly [77]. Lastly, phagocytosis of DAMPS and other cell debris leads to lysosome destabilisation and release of the enzyme cathepsin B into the cytosol to directly act on the LRR domain of NLRP3 and aid inflammasome formation (Figure 2) [78,79,80].

The subsequent formation of the NLRP3 inflammasome activates caspase-1, which cleaves and activates inactive pro-inflammatory cytokines, pro-IL-1β, and pro-IL-18 into active IL-1β and IL-18, allowing pyroptosis, a form of programmed necrosis, to occur (Figure 2) [81,82,83]. The process of pyroptosis occurs via the cleavage and separation of the N- and C-terminals of GSDMD by caspase-1 [83]. The C-terminus acts as an auto-inhibitor of the pore-forming N-terminus; however, once GSDMD is cleaved, the N-terminal forms nanopores in the cell membrane, leading to cell swelling and the release of active IL-1β and IL-18 [84,85]. Inhibition of NF-κB was established to suppress GSDMD transcription and reduce NLRP3 inflammasome-mediated pyroptosis activity within cardiomyocytes [86]. Pyroptosis can also be facilitated by the aggregation of ASC molecules into 1–2 μm ASC dimers and the recruitment of caspase-1 [87].

### 3.3. Targeted NLRP3 Inhibitors

Several small molecules examined in pre-clinical trials have shown promise as direct inhibitors of the NLRP3 inflammasome signalling pathway; however, translation into clinical use will require further assessment based on safety profiles and applicability within clinical protocols. The two most well-studied NLRP3 inhibitors that have shown therapeutic promise in IRI include MCC950 and OLT1177; this review will briefly introduce these agents but will not comprehensively discuss them as they are out of the scope of this review.

MCC950 is a sulfonylurea-containing compound that potently (IC_50_ 7.5–8 nM) binds to the ATP hydrolysis pocket of NLRP3, proximal to the Walker B motif on the NACHT domain in human monocyte-derived macrophages [88,89]. This interaction stabilises NLRP3 into an inactive conformation [90]. In a C57BL/6 mouse model of myocardial infarction (MI) involving left anterior descending artery (LAD) occlusion, 14 days of postoperative delivery of MCC950 improved ejection fraction (26.7%), reduced myocardial fibrosis (13%), and reduced myocardial NLRP3, IL-1β, IL-18, and caspase-1 whilst decreasing immune cell infiltration [91].

OLT1177 is a β-sulfonyl nitrile known to directly target and inhibit the NLRP3 inflammasome as it does not alter transcriptional regulation or activation signals involved in NLRP3 activation but decreases IL-1β and IL-18 levels as demonstrated in LPS-stimulated human monocyte-derived macrophages. [92]. In a Swiss mouse model of MI involving LAD ligation, postoperative administration of OLT1177 (6–600 mg/kg) dose-dependently reduced infarct size (between 36–62%) and preserved left ventricular fractional shortening up to 7 days post-surgery [93]. As of 2021, a Phase 1b human clinical trial conducted in patients with systolic heart failure showed that OLT1177 delivered at oral doses between 500 and 2000 mg/day for 14 days exerted no significant adverse events and improved left ventricular ejection fraction (by 5%) [94].

Recent advancements led to the development of INF4E and other chemically related non-sulfonylurea-based NLRP3 inhibitors such as INF200 [95,96]. INF4E is an acrylamide derivative considered to be a covalent inhibitor of NLRP3 and its ATPase activity. Pharmacological inhibition of the NLRP3 inflammasome with the novel potent inhibitor, INF4E, provided a cardioprotective response by activating the reperfusion injury salvage kinase (RISK) pathway and improved mitochondrial function within an ex vivo IRI rat model [97]. Pre-treatment of isolated rat hearts with INF4E (50 μM) for 20 min prior to IRI produced a 22% reduction in infarct size and improved haemodynamic function 60 min after reperfusion onset compared to controls. Penna, et al. [98] reproduced this finding, identifying RISK activation and smaller infarct size and area at risk with INF4E pre-treatment compared to the untreated group.

## 4. Gasotransmitters as Cardioprotectants

As described above, the initiation of an ischaemic insult and subsequent reperfusion induces not only an aggressive myocardial injury but also an intense cardiac inflammatory response, contributing to myocardial IRI. It is imperative that new, targeted cardioprotective agents capable of reducing the associated innate inflammatory response are introduced. A potential avenue currently gaining attention is the ability of the three gasotransmitters—carbon monoxide (CO), nitric oxide (NO), and hydrogen sulphide (H_2_S)—to reduce myocardial IRI through multiple signalling pathways, but most importantly, via a targeted response on the NLRP3 inflammasome.

### 4.1. Endogenous Carbon Monoxide

Carbon monoxide (CO) gas is commonly, and perhaps melodramatically, described as a silent killer, as exposure to high concentrations of CO can impair the O_2_-carrying capacity of the blood due to a ~220-times higher affinity of haemoglobin for CO over O_2_ [99]. Paradoxically, however, endogenous production of this gaseous signalling molecule occurs ubiquitously in mammalian cells, including pertinently in humans [100]. The rate-limiting enzymes, heme oxygenases (HOs), are responsible for this endogenous production [101]. Currently, there are two main human isoforms of HOs: HO-1, and HO-2. These enzymes catalyse the oxidative degradation of free heme to biliverdin, free iron, and CO [102,103]. HO-2 is constitutively expressed within endothelial cells and smooth muscle cells [104]. In contrast, HO-1 is inducible and is activated in response to periods of ischaemia and reperfusion and other cellular stresses, indicating a high capacity for the cardiovascular system to produce CO [105]. During these stresses, the expression of HO-1 within the heart is increased and can exert cytoprotective effects on tissue and the myocardium [106]. These effects are attributable to the production of biliverdin, an antioxidant, and more importantly, to the induction of cell signalling pathways mediated by CO [107,108].

### 4.2. Exogenous CO Delivery

While the dangers of exposure to CO are well defined, low doses of CO (<100 ppm) and molecules that release controlled amounts of CO have been demonstrated to produce therapeutic responses whilst avoiding toxic effects [109,110,111]. The initial discovery of endogenous CO production and the subsequent demonstration of the pleiotropic therapeutic effects of this gaseous molecule led to the call for pharmacological agents to be developed with the ability to systemically release controlled low concentrations of CO as potential therapeutics and as research tools. These chemical prodrugs have been classed as ‘carbon monoxide releasing molecules’ (CORMs) [112]. The first characterised CORMs consisted of a transition metal centre, such as manganese or ruthenium, surrounded by carbonyl groups, and include CORM-1 [Mn_2_(CO)_10_], CORM-2 [Ru(CO)_3_Cl_2_]_2_, and CORM-3 [Ru(CO)_3_Cl-glycinate] [112]. A non-metallo CORM-A1 [Na_2_H_3_BCO_2_] has also been synthesized as a slower CO-releasing molecule [113]. These CORMs have been widely studied within both in vivo and in vitro applications [113,114,115,116]. Whilst CORMs have been proposed to have a number of therapeutic indications, these compounds still possess a number of features that limit their use in clinical applications. Unlike gaseous CO, administration of 20 μM CORM-2 (48 h) produced both cytoprotective and cytotoxic effects in cardiomyocytes and in renal cell studies and this toxicity was associated with the ruthenium complex rather than CO [117]. Subsequent research confirmed the inherent toxicity of the metal CORMs by demonstrating CORM-3 toxicity at 500 μM within RAW 264.7 macrophages, illustrating differences in the specific sensitivity of cell lines to their toxic effects [118]. Effects attributed to the parent compound of some of these earlier CORMs rather than being mediated by the CO released have recently been described, which, combined with idiosyncratic CO release rates, limit the value of these CORMs in the study of CO-mediated mechanisms [119]. Identifying that these toxic and off-target responses relate to the prodrug structure, rather than to CO, was an important discovery and has emphasised the need for an improved molecule with minimal toxicity to serve as a CO prodrug [120,121]. Recently developed organic CO-releasing prodrugs such as oCOm-21 have demonstrated many of the same protective effects as CO but with few of the limitations demonstrated by earlier CORMs [111,122]. These new drug developments have been designed to release CO under various triggering conditions such as physiological pH and provide pharmacologically effective CO delivery to intracellular compartments.

### 4.3. Pleiotropic Effects of CO

The discovery of endogenous CO production initiated research into the physiological and therapeutic properties of this pleiotropic molecule. CO induces vasodilatory, anti-apoptotic, anti-thrombotic, anti-proliferative, and anti-inflammatory effects through p38 mitogen-activated protein kinase (p38MAPK), the phosphatidylinositol 3-kinase/protein kinase B (PI3K/Akt) and the soluble guanylate cyclase (sGC) pathway [111,123,124,125]. Investigations into the vasodilatory properties have shown that CO mediates these effects via cyclic guanosine monophosphate-dependent smooth muscle relaxation [126]. Endogenous CO generated by HO-1 was identified to suppress vascular endothelial cell apoptosis, with the mechanism being reliant on the p38MAPK pathway and NF-κB [127,128]. Furthermore, this subsequent activation of p38MAPK and NF-κB stimulates the transcription of the anti-apoptotic genes, c-IAP2 and A1, to protect vascular endothelial cells from tumour necrosis factor-alpha (TNF-α)-mediated apoptosis. Additionally, the anti-apoptotic effects of CO are not solely reliant on the p38MAPK pathway, but also on the PI3K/Akt pathway within endothelial cells [125], while anti-apoptotic studies conducted in fibroblasts show that this CO effect is mediated through sGC activation [129].

The anti-inflammatory effects of endogenous CO upregulation have also been repeatedly demonstrated [124,130,131,132]. Similarly to the anti-apoptotic response, CO can produce these beneficial anti-inflammatory effects via p38MAPK in a concentration-dependent manner within RAW 246.7 macrophages [124]. This anti-inflammatory effect of CO was associated with a modulation of the cytokine profile seen as an inhibition of LPS-induced production of TNF-α, IL-1β, and macrophage inflammatory protein 1b, whilst anti-inflammatory IL-10 production was increased. However, CO was also subsequently shown to exert concurrent anti-inflammatory effects via other mechanisms; Morse, et al. [130] found that inhalation exposure to 250 ppm CO in a mouse was capable of inhibiting the LPS-induced JNK/AP-1 pathway, while Nakahira, et al. [131] demonstrated the ability of CO to inhibit TLR signalling pathways specifically on TLR 2, 4, 5, and 9 but not on TLR3. This signal inhibition correlated with the inactivation of transcription factors such as NF-κB and interferon regulatory factor-3, which consequently, inhibited cytokine production. Additionally, Qin et al. [132] demonstrated that without the presence of nuclear factor-erythroid 2-related factor-2 (Nrf2), CO could not produce an anti-inflammatory effect in an LPS-induced inflammation mouse model. Overall, the anti-inflammatory effects of low concentrations of CO can be considered therapeutically significant and not limited to a single pathway.

### 4.4. Inhibitory Effects of CO on NLRP3

**Figure 3:**
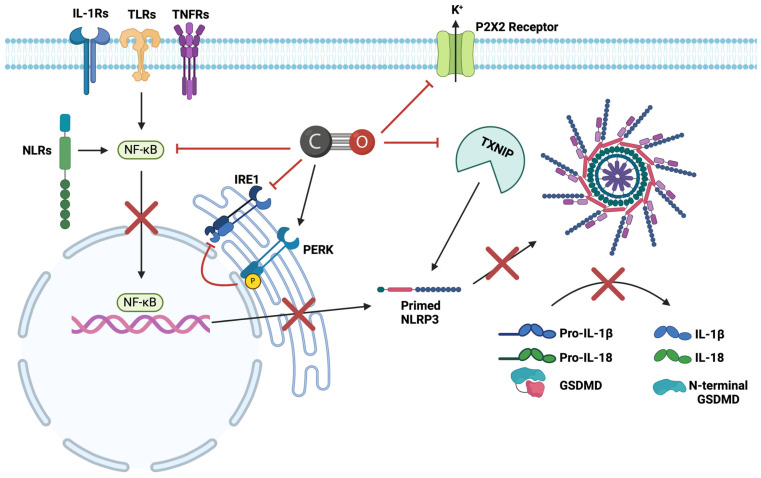
**Summary of the identified modulatory effects of CO on NLRP3 formation.** CO inhibits NF-κB to reduce transcription of NLRP3 and prevent NLRP3 formation. CO also inhibits P2X receptors to reduce K^+^ efflux. Retention of K^+^ is associated with reduced Ca^2+^-independent phospholipase A2 activation, one of the driving signals for NLRP3 activation [69]. CO inhibits TXNIP to prevent binding of NLRP3 and reduce NLRP3 formation. Lastly, CO activates PERK and inhibits IRE1 phosphorylation to reduce NLRP3 activation.

The ability of CO and CORMs to inhibit NLRP3 inflammasome formation through multiple pathways has been reported in different tissues and models. Early patch-clamp studies conducted by Wilkinson, et al. [133] indicated that both CO gas and CORM-2 can inhibit ATP-gated purinergic (P2X) receptors, specifically at P2X2 (Figure 3). As activation of P2X receptors results in reduced intracellular K^+^ (an activation signal for NLRP3), this pathway was considered to represent one possible mechanism through which CO could inhibit NLRP3 formation [51,134,135]. These studies also showed that CORM-2 was a reversible, non-competitive inhibitor at P2X4, while CO gas was not [136]. However, there is no ex vivo or in vivo evidence to support the idea that CO gas can inhibit P2X4 or P2X7 receptors in the presence of ATP, even though these receptors are regarded as the only ligand-gated ion channels regulated by CO [136,137]. Therefore, it could be assumed that the inhibition identified was due to the parent metallo carbonyl structure of the early CORMs rather than the CO molecule.

Jiang, et al. [138] elucidated an inhibitory action of CO on thioredoxin interacting protein (TXNIP) to reduce NLRP3 expression (Figure 3). While this study was performed on lung tissue, Liu, et al. [139] demonstrated that TXNIP KO in both cardiac microvascular endothelial cells and cardiomyocytes also inhibits NLRP3 activation, significantly reducing IL-1β production by ~1.7 fold and suggesting a potential, indirect, inhibitory action for CO on NLRP3. Additionally, the researchers also identified that within a mouse myocardial IRI model, infarct size, measured as a ratio of infarct area to total area at risk, was significantly reduced by ~18%, and left ventricular ejection fraction, used as a measure of cardiac function, was restored. Zhou et al. [140] showed that ROS generation from NLRP3 activators such as uric acid crystals is required to release TXNIP from oxidised thioredoxin, which allows the binding and activation of NLRP3. Interestingly, inositol-requiring enzyme 1 (IRE1) has been shown to interact with TXNIP/NLRP3 signalling, indicating a significant modulatory role in NLRP3 inflammasome activation [141]. CO was shown to suppress IRE1 phosphorylation by inducing protein kinase R-like endoplasmic reticulum kinase phosphorylation (PERK) [142,143]. This finding is further backed by Zheng et al. [144] who reported that administering CORM-3 (8 mg/kg/day, i.v.) to rats for 7 days reduces IRE1 phosphorylation and decreases NLRP3 inflammasome production and pyroptosis in a spinal cord injury model (Figure 3). Lastly, Zhang et al. [145] proposed that CO interacts with NF-κB signalling to prevent upregulation of NLRP3 and pro-IL-1β. These authors have, however, indicated that CO could also inhibit the activation stage as the addition of CORM-3 after LPS priming reduced active IL-1β and IL-18 production (Figure 3). CO, therefore, has a broad inhibitory effect on the priming stage of NLRP3, preventing NLRP3 upregulation and the subsequent inflammation. To date, no research has investigated the ability of CO to directly inhibit NLRP3 inflammasome formation within myocardial IRI.

### 4.5. Endogenous Nitric Oxide

NO was first identified as an endothelium-derived relaxing factor by Palmer et al. [146] and found to be endogenously synthesised in endothelial cells through the degradation of L-arginine by NO synthase (NOS) [147]. Current NOS family isoforms identified include endothelial NOS (eNOS), neuronal NOS (nNOS), and inducible NOS (iNOS) [148,149,150]. eNOS is constitutively expressed within endothelial cells and is widely accepted as the primary source of NO present in the vasculature, contributing to the regulation of vascular tone, systemic and pulmonary vascular resistance, inhibition of platelet aggregation, and leukocyte adhesion [151,152,153,154]. nNOS was preliminarily identified within the brain and autonomic nerves owing to its pivotal role in controlling cerebral functions and modulating glutamatergic/GABAergic neurotransmission [155,156]. However, nNOS has also been identified to be constitutively expressed within the vascular endothelium and can contribute towards NO production to modulate cerebral blood flow and systemic arterial pressure [157,158]. In contrast, iNOS is not constitutively expressed; instead, its expression is regulated via NF-κB signalling during pro-inflammatory and oxidative stress conditions [159]. This inducible isoform is most commonly present within circulating neutrophils and macrophages but is also upregulated within cardiomyocytes in response to pro-inflammatory cytokines [148]. Upon activation, iNOS activity produces micromolar concentrations of NO that can both induce as well as attenuate, upon elevation, pro-inflammatory responses through a biphasic regulatory effect on NF-κB [160]. In contrast, NO production via eNOS and nNOS can produce nanomolar concentrations of NO, which have proven to induce cytoprotective effects [161]. Importantly, all of these NOS isoforms have been identified within the whole heart and can contribute to NO production during myocardial IRI [162].

### 4.6. Exogenous NO Application

The use of gaseous NO inhalation in both therapeutic and experimental research settings is greatly hindered by delivery constraints, unlimited diffusion, and lack of compartmentalisation to target tissues, and by the extremely short (0.05–2 s) half-life of this avidly reactive radical in biological conditions [163]. These characteristics have warranted the production of pharmacological agents that have the capacity to release NO or upregulate endogenous NO production to improve site-specific delivery of active, therapeutic levels of this gaseous transmitter. These compounds include NO donors such as organic nitrates, diazeniumdiolates, S-nitrosothiols, furoxans, metal nitrosyl compounds, and nitrobenzenes [164]. For decades, these compounds have been utilised both clinically and experimentally to further assess the therapeutic capabilities of this gaseous molecule and have provided some promising results [165,166]. However, it should be noted that the free radical nature of NO can produce a variety of toxic effects that pose a barrier to the safe therapeutic use of this molecule. NO is able to readily interact with superoxide, with the potential to produce a 100-fold increase in levels of the potent oxidant, pro-inflammatory peroxynitrite for every 10-fold increase in both NO and superoxide [167] and induce cardiomyocyte injury [168,169].

### 4.7. Pleiotropic Effects of NO

Both the endogenous synthesis and exogenous application of NO have been shown to provide cardioprotection during myocardial IRI [160,161,167]. Similar to CO, NO has a number of valuable pleiotropic actions, including anti-inflammatory, anti-apoptotic, and, most notably, vasodilatory attributes associated with the expression of NOS within the vasculature [151,170,171]. This vasodilatory mechanism involves a NO-mediated activation of sGC leading to the formation of cGMP similar to that produced in response to CO. NO has, however, been reported to avidly bind the heme moiety on sGC to produce a 200-fold increase in cGMP compared to CO, which, potentially because of the fast dissociation of CO from heme, results in CO exerting relatively low (4-fold) increases in sGC activation [172,173].

The ability of NO to reduce apoptosis by inducing the phosphorylation, and hence activation of ERK1/2 resulting in a reduction in caspase-3 activity, was demonstrated using the NO donor S-nitroso-N-acetylpenicillamine (SNAP) at 10 µM in a mouse model of myocardial IRI [170]. This protection could be reversed by the addition of the selective ERK1/2 inhibitor, U0126, indicating that the anti-apoptotic effect induced by NO is mediated via the ERK1/2 pathway [170]. It should be noted that NO has also been reported to induce apoptosis through the activation of BAX, BAK, and caspase-9, allowing cytochrome c to be released from the mitochondria, thereby activating the intrinsic apoptotic pathway [174].

Enzymatic activity by iNOS produces high (micromolar) levels of NO, which can promote the pathogenesis of inflammatory disorders such as sepsis, rheumatoid arthritis, and inflammatory bowel disease [175,176,177]. Conversely, NO production at low (nanomolar) concentrations has been recognised to promote an anti-inflammatory effect [160]. By applying both in vitro and in vivo experimental protocols, Lee et al. [178] found that increased NO release through eNOS overexpression, NO donor administration, or endothelial–macrophage co-culture can induce macrophage polarisation. This polarisation away from the pro-inflammatory M1 towards an M2 phenotype is indicated to occur endogenously through the downstream overexpression of vasodilator-stimulated phosphoprotein within macrophages. Confirmation of the physiological significance of NO in repressing M1 activation was also observed in macrophages co-cultured with eNOS-depleted aortic endothelial cells and in vasodilator-stimulated phosphoprotein-deficient transgenic mice [178]. Niedbala et al. [179] also identified the significance of NO-induced proliferation of CD4^+^ CD25^−^ T cells into regulatory T cells in inflammatory tissue, allowing these cells to produce IL-10 and IL-4. This proliferative induction of regulatory T cells by NO (200–400 nM) has been paradoxically suggested to be dependent on p53 enhancing IL-2 and OX40 synthesis. In a separate study, Niedbala et al. [180] demonstrated that NO could inhibit the expression of the aryl hydrocarbon receptor in response to environmental stimulants/pollutants, thereby suppressing the function and proliferation of human Th17 cells under autoimmune conditions.

### 4.8. Inhibitory Effects of NO on NLRP3

**Figure 4:**
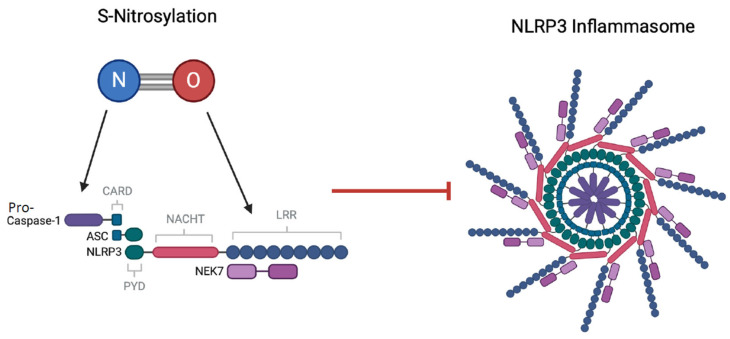
**S-nitrosylation inhibition of NLRP3 Inflammasome activation.** Post-translational covalent attachment of an NO group (to a cysteine thiol) occurs both at the LRR domain of NLRP3 and on pro-caspase-1 to prevent NLRP3 inflammasome formation.

Unlike CO, treatment with either SNAP or the iNOS inhibitor L-NMMA has shown that NO molecules can directly inhibit the NLRP3 inflammasome through S-nitrosylation (Figure 4) [181,182]. S-nitrosylation is a post-translational modification mechanism where NO groups are covalently added to cysteine protein thiols, regulating their function [183]. Hernandez-Cuellar et al. [181] used a biotin-switch technique to identify that S-nitrosylation occurred both at the C-terminus of the NLRP3 protein and on caspase-1, preventing early NLRP3 inflammasome formation (Figure 4). Both caspase-1 and the C-terminus of the NLRP3 inflammasome (the LRR domain) contain cysteine residues, providing multiple functional targets for S-nitrosylation to occur [184]. Thiol sidechains contained within these active sites on caspase-1 can be S-nitrosylated, attenuating the proteolytic function of caspase-1 (Figure 4) [185]. Collective evidence by Mao et al. [182] and Mishra et al. [186] further demonstrated that S-nitrosylation is required to occur directly on both NLRP3 and caspase-1 in order to suppress the formation of IL-1β, as S-nitrosylation of caspase-1 alone is insufficient. These findings have consistently shown the ability of NO to inhibit the activating stage of the NLRP3 inflammasome within macrophages, although this mechanism is yet to be directly investigated within the myocardium. Both exogenous and endogenous-derived NO have repeatedly demonstrated cardioprotection in IRI [187,188,189] and, pertinently, to reduce myocardial inflammation [190]. These findings lead to the suggestion that the mechanism through which NO reduces inflammation during myocardial IRI is afforded by a direct inhibitory effect on NLRP3 inflammasome activation, although further research will be required to confirm this.

### 4.9. Endogenous Hydrogen Sulphide

Hydrogen sulphide (H_2_S) is the third endogenous gasotransmitter shown to have beneficial physiological properties [191,192]. The oxidation state of the sulphur atom at −2 allows H_2_S to become oxidised at physiological pH and react with metal centres such as iron in heme, as well as oxidised thiol products, to form persulphides. H_2_S is predominantly synthesised through desulphhydration of biomolecules such as L-cysteine and homocysteine via cystathionine β-synthase, 3-mercaptopyruvate sulfotransferase, and cysteine aminotransferase enzymes located within the central nervous system, and by cystathionine γ-lyase (CSE) predominately found within the cytosol of cardiovascular, hepatic, and renal tissues [193,194,195].

### 4.10. Exogenous H_2_S Application

The identification of endogenous H_2_S involvement in a range of cytoprotective effects has spurred research into the use of both gaseous and synthetic H_2_S-releasing agents in experimental and therapeutic applications. Recognition, however, of the noxious nature of H_2_S gas and that high (100 ppm) concentrations can produce adverse effects [196] such as mitochondrial cytochrome c inhibition [197], have tempered the use of gas inhalation as a therapeutic application. These considerations have consequently warranted the development of a range of pharmacologically effective agents that can endogenously increase localised concentrations of H_2_S. To date, experimental studies have mainly employed either inorganic salts such as sodium hydrosulphide (NaHS) and sodium sulphide (Na_2_S) or organic compounds such as the naturally occurring, fast-releasing diallyl trisulphide and the slow-releasing diallyl disulphide donors [198,199]. Classified according to the mechanism through which they increase H_2_S levels, these synthetic organic donors include hybrid molecules containing a H_2_S donating moiety, spontaneously releasing H_2_S donors, and compounds which serve as substrates for H_2_S-generating enzymes [200]. One leading slow-releasing H_2_S donor in the clinical setting has been the orally available SG1002, which completed phase I clinical trials for safety and tolerability in patients with heart failure (seven subjects) [201]. While phase II trials are yet to be performed with SG1002, several preclinical studies have provided strong support for the use of H_2_S as a cardioprotectant [202,203,204]. Unfortunately, these experimental studies have provided limited data on the long-term impact of the drug, and further research using a large animal model will be required before pursuing the use of these agents within the clinic [205].

### 4.11. Pleiotropic Effects of H_2_S

There are consistent findings that augmentation of H_2_S through exogenous and endogenous mechanisms provides vasorelaxant, anti-apoptotic, anti-oxidative, and anti-inflammatory properties within pathologies such as endothelial dysfunction, myocardial fibrosis, and hypertrophy, leading to the benefits shown in myocardial, hepatic, and renal IRI [203,206,207,208,209]. Zhao et al. [210] originally reported that exogenous administration of H_2_S-solubilised gas induced vasorelaxation with an IC_50_ of 125 ± 14 μM within phenylephrine-precontracted rat aortic tissues, and that this effect was also obtained through bolus intravenous delivery of H_2_S in vivo. This H_2_S-induced response was inhibited both in vivo and ex vivo by the addition of glibenclamide, demonstrating that this vasorelaxant effect is mediated by ATP-dependent K^+^ channels (K_ATP_). Lee et al. [211] further indicated that the vasodilatory effects of H_2_S within rat aortic rings were also partly regulated by a decrease in intracellular pH via the Cl^−^/HCO_3_^−^ exchanger. There are discrepancies within the literature, however, with some reports indicating a smooth muscle vasoconstrictive effect produced by H_2_S. Lim et al. [212] reported that the exogenous donor, NaHS (yielding 3–30 µM H_2_S) induced vasoconstriction via a decrease in NO production and an attenuation of forskolin-induced cAMP accumulation. This inhibition of cAMP production by H_2_S has also been noted in cardiac myocytes [213,214]. Whilst these articles have identified vasoconstrictive responses to H_2_S, the majority of the literature largely supports a beneficial vasodilatory effect, which has also been consistently reported in vivo [215,216,217].

The anti-apoptotic effects of H_2_S were identified within a rat LAD ligation myocardial IRI model where pre-treatment with 3 mg/kg NaHS prevented caspase-9 activation and increased Bcl-2 expression [203]. Examination of the area of myocardium at risk after IRI found that pre-treatment with NaHS had significantly decreased cardiomyocyte apoptosis from 37 ± 4% to 17 ± 2% and caspase 9 activity from 34 ± 2% to 20 ± 2%. Abrogation of this protective effect with 5-hydroxydecanoate (5-HD), a K_ATP_ channel blocker, demonstrated that these effects were secondary to the opening of mitochondrial K_ATP_ channels. This anti-apoptotic effect was also demonstrated with much lower (0.2 and 0.4 μmol/kg) doses of NaHS in a cerebral rat IRI model where treatment increased anti-apoptotic Bcl-2 expression whilst decreasing pro-apoptotic Bax expression [218].

H_2_S is recognised as an important player in providing cardioprotection during IRI. Sivarajah et al. [219] found that upregulation of endogenous H_2_S through cystathionine-γ-lyase occurred during cardiac ischaemia and reperfusion, providing cardioprotection and limiting the extent of myocardial structural injury. The cardioprotective effects afforded by H_2_S were abrogated by the addition of 5-HD, supporting the importance of K_ATP_ channels in H_2_S signalling. Furthermore, Johansen et al. [206] showed that NaHS (0.1–10 μM) produced a concentration-dependent reduction in infarct size in ex vivo Langendorff-perfused rat hearts subjected to IRI. Comparatively, in vivo administration of 50 μg/kg of NaHS at the time of reperfusion in mice subjected to 30 min of left ventricular ischaemia significantly reduced cardiac infarct size from 47.9 ± 2.9% to 13.4 ± 1.4%, reduced cardiac inflammation, and preserved mitochondrial function 24 h post reperfusion onset [220].

**Figure 5:**
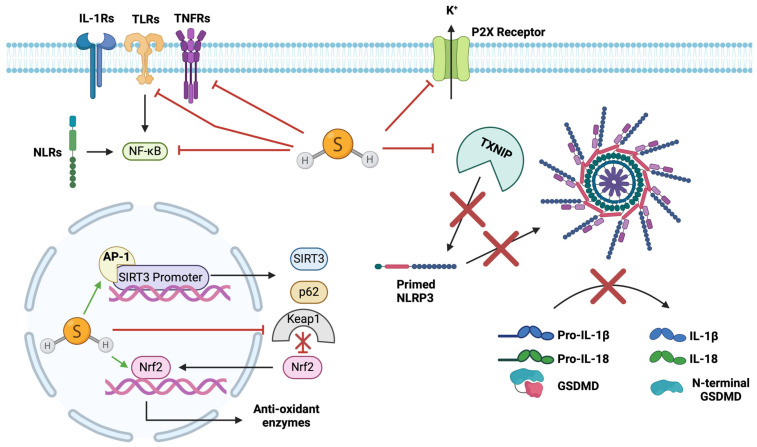
**Summary of the modulatory effects of H_2_S on NLRP3 inflammasome formation and activation.** H_2_S inhibits several proteins, including NF-κB to reduce NLRP3 transcription, TNFRs to reduce NF-κB activation, TXNIP to reduce binding to NLRP3, P2X receptors to reduce K^+^ efflux, and Keap1 to promote translocation of Nrf2 into the nucleus. H_2_S activates Nrf2 to upregulate antioxidant enzymes and AP1 to upregulate SIRT3 promotor and increase SIRT3 and p62 levels to reduce NLRP3 activation. Green arrows indicate the activating response of H_2_S.

The anti-inflammatory capabilities of H_2_S have been consistently demonstrated to act through similar pathways to those activated by CO [203,218,221,222,223]. Increased phosphorylation of both p38 MAPK and NF-κB has been identified following NaHS administration in IRI studies conducted in gastric epithelium and myocardium, resulting in attenuated neutrophil infiltration and ICAM-1 and PMN accumulation within areas of tissue at risk [203,224]. Pre-treatment of these gastric epithelial cells with 100 μM NaHS after IRI also resulted in an attenuation of JNK phosphorylation [224]. A reduction in IL-1β, IL-6, and TNF-α levels associated with an inhibition of NF-κB was identified after treatment with NaHS in a rat myocardial IRI model [225]. Furthermore, H_2_S has also been recognised to strongly interact with Nrf2 signalling, providing significant cardioprotective effects [226,227]. Nrf2 is the primary cellular defence against oxidative stress and is normally restricted to the cytoplasm by Keap1 [228]. H_2_S has been identified to interact with this association by S-sulphhydration of cysteine residues on Keap1, specifically cysteine-151, allowing Nrf2 to enter the nucleus and begin transcription of antioxidant enzymes as well as, interestingly, the CO-producing enzyme, HO-1 (Figure 5) [227,229].

### 4.12. Inhibitory Effects of H_2_S on NLRP3

The anti-inflammatory capabilities of H_2_S have been determined to involve interactions upon NLRP3 inflammasome signalling through a variety of mechanisms: H_2_S induces a post-translational modification, similar to NO, wherein an additional sulphur molecule is added to thiol groups of cysteine; this event is termed S-sulphhydration [230]. Lin et al. [231] identified this mechanism on c-Jun, the subunit of activator protein-1, at Cys269 using a biotin switch assay within macrophages. This modification enhanced binding of the activator protein-1 (AP-1) to the SIRT3 promoter, increasing transcription of SIRT3 and p62, which subsequently inhibited NLRP3 inflammasome formation (Figure 5). Whilst this event was identified within macrophages, it has been consistently recognised that macrophage NLRP3 inflammasome activation plays a role in cardiovascular disease [232,233,234]. Furthermore, H_2_S has also previously been shown to interact with and enhance AP-1 binding and SIRT3 expression within endothelial cells, inducing a vasoprotective effect, providing further insight into its capability to interact with NLRP3 inflammasome activation within the cardiovascular system (Figure 5) [209].

Zhao et al. [235] reported that the induction of intracerebral haemorrhage producing neurological deficit and brain oedema in a rat model temporarily decreased cystathionine-β-synthase expression and endogenous H_2_S levels within the striatum lasting 48 h. This attenuation of endogenous H_2_S was associated with a significant increase in P2X7 receptor expression and NLRP3 inflammasome component levels. Administration of either NaHS or the cystathionine β-synthase agonist *S*-adenosyl-L-methionine one day after intracerebral haemorrhage resulted in a significant reduction in P2X7 receptor, NLRP3, and IL-1β levels (Figure 5). Interestingly, both endogenous and exogenous H_2_S have also been shown to suppress hyperglycaemia-induced TXNIP expression in a type-1 diabetic cardiomyopathy rat, resulting in a decreased response in NLRP3 complex activation (Figure 5) [236,237]. This inhibition of TXNIP expression had also been previously noted following administration of the other gaseous signalling molecule, CO, in lung tissue [138]. Huang et al. [238] had shown that pre-treatment with 400 μM NaHS offered protection to H9c2 cardiomyocytes subjected to high-glucose-induced cardiotoxicity. The addition of NaHS prevented high-glucose-induced expression of the NLRP3 inflammasome via direct suppression of the TLR4/NF-κB pathway [239,240] (Figure 5). This has been further demonstrated more recently in a mouse model of LPS-induced myocardial injury, whereby 50 μmol/kg of NaHS downregulated TLR4 and NLRP3 expressions by 64% and 31%, respectively, and enhanced ventricular function by 0.19-fold [241]. Other models have been used to show that H_2_S can also prevent oleic acid-induced suppression of the AMPK/mTOR pathway, promoting autophagy and inhibiting the NLRP3 inflammasome [242]. As previously stated, H_2_S has important interactions with Nrf2 to induce cytoprotective effects, and this interaction plays a key role in inhibiting NLRP3 inflammasome priming and activation [243,244]. This interaction between Nrf2 and H_2_S has been shown to attenuate renal IRI [245] and while this pathway has not been investigated in myocardial IRI, it can be hypothesised that the same protective response will occur.

## 5. Limitations of the Current Literature

Inflammation is often viewed as a double-edged sword, destructive if hyperactive, but favourable during low activity. Evidence of the key role played by the NLRP3 inflammasome in cardiac injury during reperfusion has been demonstrated in hearts from NLRP3/ASC double KO C57BL/6 mouse models subjected to ex vivo IRI [34]. In comparison to wildtype C57BL/6 mice, cardiac infarct size was significantly reduced in the knockouts by ~17% and haemodynamic contractile function was better preserved. Most of the current literature states that the presence of the NLRP3 inflammasome during cardiac ischaemia and reperfusion is detrimental and exacerbates the degree of tissue damage; however, there are reports indicating that the formation of the inflammasome can, in fact, be cardioprotective.

Zuurbier et al. [246] identified a cardioprotective role of NLRP3 in an NLRP3/ASC KO mouse model. This study indicated that without NLRP3, IL-6 levels decreased, resulting in a loss of the cardioprotective IL-6/signal transducer and activator of transcription 3 signalling pathway and hence a loss of cardioprotective ischaemic preconditioning. This study did not, however, directly measure infarct size, but relied on lactate dehydrogenase release as a marker of tissue damage. Controversially, Sandanger et al. [247] also suggested that NLRP3 may paradoxically be cardioprotective during in vivo cardiac IRI, a finding that appears to conflict with earlier findings by the same group in an ex vivo perfused heart model [34]. By conducting in vivo myocardial IRI in an NLRP3/ASC KO mouse model, Sandanger et al. [247] reported that cardiac infarct size based upon cardiac troponin measurements increased and RISK activation was lost in the knockout animals. There are several reservations, however, with both the KO model used and the study, chief of which is that IL-1β mRNA, rather than protein expression, was used to indicate inflammasome activation. Interestingly, Hollmann and Zuurbier [248] proposed that in the absence of surgical stressors such as tissue injury, there are inadequate resident levels of NLRP3 within the myocardium to actually contribute toward myocardial IRI. In their study, Hollmann and Zuurbier [248] used an in vivo closed-chest model with the ligature left on the left anterior descending coronary artery for 10 days in situ prior to inducing IRI in order to eliminate surgical stressors as a potential factor for inducing NLRP3 expression. The inclusion of an open-chest surgical model to directly compare against the closed-chest model in the same study identified increased expression of both cardioprotective IL-6 and TNF-α and the NLRP3 inflammasome.

## 6. Concluding Remarks

This review provides an overview of the signalling modulatory effects of the three gasotransmitters CO, NO, and H_2_S, focusing on the summary of published evidence supporting the value of these molecules as cardioprotective agents. The data gathered strongly suggests that all three gasotransmitters can exert a valuable anti-inflammatory effect by downregulating NLRP3 inflammasome activation in whole animal models. How effective NLRP3 inactivation remains following pharmacological intervention with any agent remains to be determined. These promising results still await validation in larger animal model studies. Currently, research in the field is lacking, and further work is required to identify precisely how these molecules, particularly CO and H_2_S, interact with intermediates involved in NLRP3 inflammasome activation to induce their cardioprotective effects. We have highlighted the limitations of the current data on the role of the NLRP3 inflammasome in myocardial IRI. However, the evidence predominantly supports that NLRP3 inflammasome activation has a detrimental role, further promoting the adverse events associated with myocardial IRI. Given the pleiotropic and largely beneficial cardioprotective effects reported in the literature, strategies to develop tunable gasotransmitter-releasing pharmacological agents should be pursued to maintain the NLRP3 inflammasome in its inactive form.
